# Performance of a modified and intermittently operated slow sand filter with two different mediums in removing turbidity, ammonia, and phosphate with varying acclimatization periods

**DOI:** 10.1016/j.heliyon.2023.e22577

**Published:** 2023-11-20

**Authors:** Nurina Fitriani, Ledy Theresia, Timothy Tjahja Nugraha O'Marga, Setyo Budi Kurniawan, Agus Supriyanto, Siti Rozaimah Sheikh Abdullah, Luuk C. Rietveld

**Affiliations:** a Department of Biology, Faculty of Science and Technology, Universitas Airlangga, Kampus C UNAIR, Jalan Mulyorejo, Surabaya 60115, Indonesia; b Laboratory of Algal Biotechnology, Centre Algatech, Institute of Microbiology of the Czech Academy of Sciences, Opatovický mlýn, Novohradská 237, 379 81 Třeboň, Czech Republic; c Department of Chemical and Process Engineering, Faculty of Engineering and Built Environment, Universiti Kebangsaan Malaysia, 43600 UKM Bangi, Selangor, Malaysia; d Department of Water Management, Faculty of Civil Engineering and Geosciences, Delft University of Technology, Stevinweg 1, CN Delft 2628, Netherlands

**Keywords:** biofilm, Filtration, *schmutzdecke*, Wastewater, Water treatment

## Abstract

The present study investigated the utilization of blood clam shells as a potential substitute for conventional media, as well as the influence of the acclimation time on the efficacy of an intermittent slow sand filter (ISSF) in the treatment of real domestic wastewater. ISSF was operated with 16 h on and 8 h off, focusing on the parameters of turbidity, ammonia, and phosphate. Two media combinations (only blood clam shells [CC] and sand + blood clam shells [SC]) were operated under two different acclimatization periods (14 and 28 d). Results showed that SC medium exhibited significantly higher removal of turbidity (*p* < 0.05) as compared to CC medium (45.99 ± 26.84 % vs. 3.79 ± 9.35 %), while CC exhibited slightly higher (*p* > 0.05) removal of ammonia (23.12 ± 20.2 % vs. 16.77 ± 16.8 %) and phosphate (18.03 ± 11.96 % vs 13.48 ± 12 %). Comparing the acclimatization periods, the 28 d of acclimatization period showed higher overall performances than the 14 d. Further optimizations need to be conducted to obtain an effluent value below the national permissible limit, since the ammonia and phosphate parameters are still slightly higher. SEM analysis confirmed the formation of biofilm on both mediums after 28 d of acclimatization; with further analysis of schmutzdecke formation need to be carried out to enrich the results.

## 1 Introduction

In developing countries, including Indonesia, domestic discharges account for approximately 85% of water pollution [1]. Colloidal and suspended particle organic compounds generated by anthropogenic activities can manifest in aquatic environments, impeding the penetration of light and resulting in water turbidity [2,3]. The use of detergents, soap, and shampoo increases the phosphate concentration and pH of receiving water bodies [4,5]. Urine, defecation, and industrial wastewater are the primary sources of ammonia [6]. Eutrophication can be induced by the presence of elevated levels of nitrogen and phosphate in a water body, resulting in the proliferation of plants and algae and impeding the penetration of sunlight into the water column. Consequently, this phenomenon can upset the ecological balance and functioning of aquatic organisms [[7], [8], [9], [10]].

Intermittent slow sand filter (ISSF) can be, apart from all types of conventional wastewater treatment technologies, an affordable option to improve water quality by treating domestic wastewater before discharge [[11], [12], [13]]. ISSF is commonly accompanied by a roughing filter (RF) as a pretreatment unit [14]. ISSF does not require chemicals (coagulants) and can be easily used on a domestic scale [13,15]. In addition, it has good removal efficiencies for turbidity, TSS, and coliform [16,17]. During the process of slow sand filtration, a biofilm layer, measuring several millimeters in thickness, develops on the surface of a fine sand layer, commonly referred to as schmutzdecke [18]. The composition of the schmutzdecke layer mostly consists of deposits generated by microorganisms [19]. The formation of biofilm layers is highly related to the acclimatization period [20].

Previous research mentioned that the acclimatization period played an important role in the formation of biofilms, which later also formed schmutzdecke [21,22]. The appropriate acclimatization period provided a good foundation for biofilm to be biologically active and effectively degrade pollutants [23]. In addition to that, the biologically active and stable biofilm was showing a higher resistance to the incoming toxins than the non-acclimatized one [23]. Biofilm and schmutzdecke layers that formed after a sufficient acclimatization period create tighter gaps between the medium, allowing higher contact time between bacteria and pollutants, which may increase the overall performance of the slow sand filter [24,25]. Current researches are also focusing on the utilization of alternative mediums in ISSF, especially low-cost materials, to substitute the common sand medium [[26], [27], [28]]. Agricultural waste (converted into biochar) and any waste materials have different characteristics, such as grain size and chemical characteristics, which may benefit the performance of a slow sand filter [27]. Additionally, waste materials are cheap, abundant, and locally available [26,29].

Research on ISSF, particularly in tropical countries like Indonesia, is currently not new, but essential as an alternative to conventional water and wastewater treatment, which utilize many chemicals; however, this area is yet to be explored further. The aim of this study was to assess the effectiveness of ISSF in wastewater purification and to examine the presence of a schmutzdecke layer through microscopy analysis. This investigation explored the impact of different medium compositions and the significance of an acclimatization period. Additionally, the study investigated the potential of utilizing clam shell waste as an alternative medium and examined the effects of varying acclimatization periods. The presented results are expected to shed light on the influence of waste materials (especially blood clam shells) used as alternative mediums and the acclimatization period on the performance of ISSF in removing pollutants from wastewater.

## 2 Materials and methods

### 2.1 Experimental set-up

**Fig. 1:**
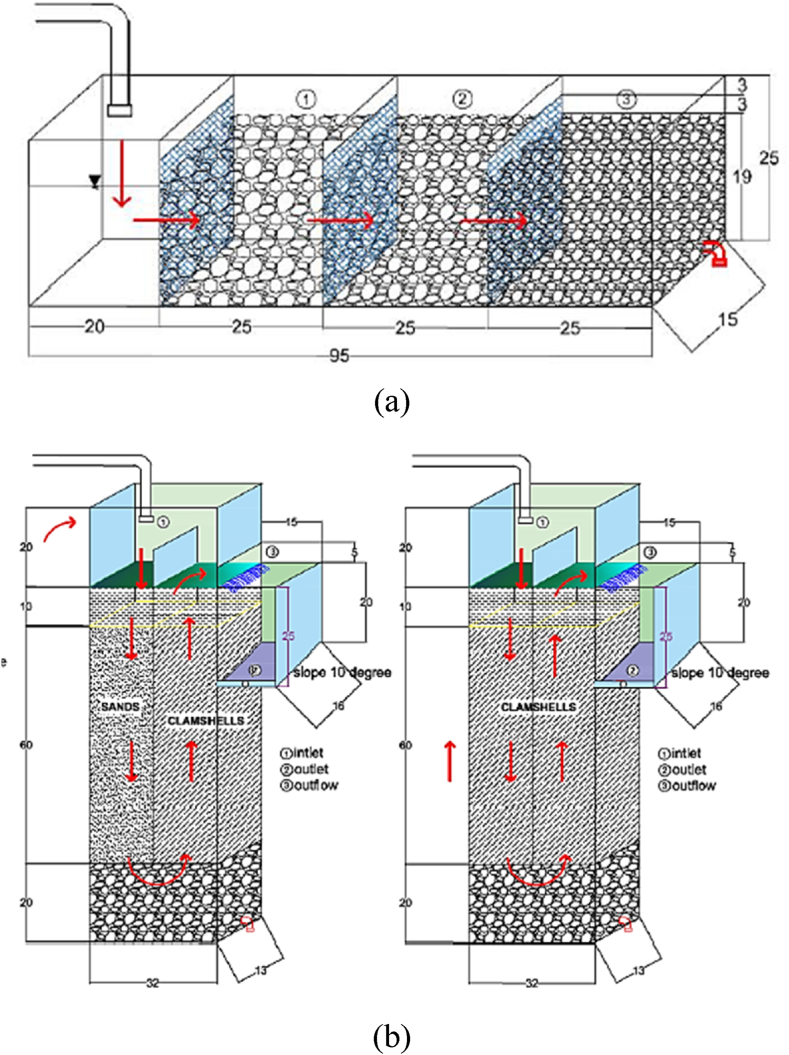
Configuration of (a) horizontal roughing filter and (b) intermittent slow sand filter.

A horizontal roughing filter (HRF) was made of an acrylic board of 10 mm thickness and served as a pre-treatment unit, while two intermittent slow sand filter (ISSF) units were constructed from 10 mm thick glass. HRF was made of 3 compartments (total effective volume of 0.03 m^3^) in which the first compartment was filled with coarse gravel (∼⌀ 3 cm), the second compartment filled with medium gravel (∼⌀ 2 cm), and the last compartment was filled with small gravel (∼⌀ 1 cm) [30] (Fig. 1a). For the ISSF, two medium combinations were used (fine sand + blood clam [SC] and only blood clam [CC]) with a total effective volume of 0.04 m^3^ (Fig. 1b). Blood clam (*Anadara granosa*) shells were proven to be good mediums and abundantly available in the research area [15,31,32]. The blood clams were subjected to a series of preparation steps, including cleaning, mechanical pulverization, and sifting, resulting in effective sizes (ES) of 0.25 mm and 0.42 mm. Real domestic wastewater was collected from Surabaya's Rusunawa Penjaringan Sari and Rusunawa Keputih wastewater treatment plants' (WWTP) inlets and mixed equally (ratio of 50:50).

### 2.2 Acclimatization and reactor operation

The acclimation stage involved intermittent exposure of the ISSF to pretreated water for a duration of 16 h per day, with a flow velocity of 0.05 m/s. Following the completion of the filtering phase, the ISSF was activated by initiating a continuous water outflow for a duration of about 16 h, with alternating periods of 8 h on and 8 h off. This operational procedure facilitated the removal of pollutants through the designated pollutant removal process. Acclimatization was conducted under two different conditions (acclimatization I for 14 days and acclimatization II for 28 days) based on our previous study for the systems (SC and CC) to reach steady state [13,19]. The system was run for main research for 10 days after each different acclimatization period, with a total restart of the system after each run. The schmutzdecke formation was observed after each acclimatization period, by observing the slimy structure formation on the top of the medium.

### 2.3 Water sample analysis and initial wastewater characteristics

The specimen was obtained using a glass receptacle and expeditiously sent to the laboratory within a refrigerated container for further examination. The turbidity parameter was measured on-site using a turbidimeter, namely the Thermo Scientific Eutech TN-100 Waterproof Turbidimeter manufactured in Germany. Based on the specifications outlined in Indonesian National Standard No. 06-6989.30-2005, the quantification of ammonia concentration was carried out utilizing the phenate technique in conjunction with spectrophotometry. In accordance with Indonesian National Standard No. 06-6989.31-2005, the phosphate concentration was measured using the lead chloride method and spectrophotometry. The initial turbidity, ammonia, and phosphate concentrations of the mixed effluent were 67.35 NTU, 20.89 mg/L, and 6.12 mg/L, respectively. The calculation of each parameter removal was conducted by subtracting the initial HRF inlet value from the ISSF outflow value following 16 h of treatment.

### 2.4 Statistical analysis

The study responses were individually and statistically assessed using an ANOVA test with a 2FI model and a significance threshold of *p* < 0.05. In cases where the data did not adhere to the usual requirements for ANOVA analysis, the Welch analysis method was employed to investigate and analyze the data. The Duncan post-hoc analysis was employed in order to determine the statistical significance of the data [33,34].

### 2.5 SEM-EDX analysis

Biofilm-grown filter media samples (SC and CC) weighing approximately 1–2 g were collected using a sterile-modified spatula. The specimens were carefully transferred into a sterile container and thereafter subjected to air-drying under ambient conditions. The specimens were affixed to specimen receptacles using carbon tape. The specimens underwent a gold (Au) coating process under a vacuum environment utilizing the COXEM SPT-20 Ion Sputter Coater [[35], [36], [37]]. Subsequently, they were examined using the HITACHI FlexSEM 1000 VP-SEM [19]. The elemental composition of the media was performed using INCA-EDS software equipped with the equipment.

## 3 Results and discussions

### 3.1 The influence of filter media on the reduction of turbidity, ammonia, and phosphate removals

The results of the statistical analysis of turbidity indicated that it was normally distributed but not homogeneous. The Welch analysis revealed a statistically significant difference between the inlet, CC outlet, and SC outlet. However, only filtration through the SC media reduced the average turbidity value. The inlet turbidity was 5.66 ± 3.85 NTU, with the lowest value being 1.18 NTU and the highest value being 16.13 NTU. CC outlet sample turbidity measured 12.62 ± 6.8 NTU, while SC outlet sample turbidity measured 2.53 ± 1.53 NTU.

The results of the statistical analysis of ammonia indicate that its concentration was normally distributed and homogeneous. There were significant differences between the ammonia concentrations in the inlet, CC outlet, and SC outlet, as determined by ANOVA. The results of the Duncan test revealed that the average ammonia concentration in SC and CC was substantially lower than the average concentration at the inlet. The ammonia content in the input sample was determined to be 29.641 ± 9.511 mg/L, with a minimum recorded value of 14.240 mg/L and a maximum recorded value of 47.156 mg/L. The concentration of ammonia in the CC sample was determined to be 22.179 ± 6.986 mg/L, but in the SC sample, it was found to be 26.081 ± 9.418 mg/L. The lack of a significant difference in discharge concentration between the two media suggests that the addition of sand to the blood clam medium had no effect on the removal of ammonia. Biological removal of ammonia occurs by nitrification and denitrification, in which two different oxic conditions are required for this mechanism to occur continuously or simultaneously [38,39]. The growth of biofilm and schmutzdecke layers in ISSF creates smaller gaps between mediums, which may create a decrease in dissolved oxygen concentration, creating an anerobic condition [40]. The gaps might be even tighter after the introduction of sand in medium combinations. In the biological removal of ammonia, nitrification should occur before the denitrification process, and this oxidation process is facilitated by nitrifying bacteria under aerobic conditions [41,42]. The reduction of dissolved oxygen caused by tighter gaps between mediums might be the reason why only a small removal of ammonia was obtained in all systems.

The results of the statistical analysis of phosphate indicate that its concentration was normally distributed and homogeneous. The ANOVA demonstrated a statistically significant difference in the levels of phosphate concentration among the inlet, CC outlet, and SC outlet. The findings from the Duncan test indicated that the discharge values of the SC and CC were much lower compared to the inlet values. However, there was no statistically significant distinction seen between the different media employed. The influence of media on phosphate reduction was seen, however, no statistically significant distinction was found between the CC and SC media. The phosphate concentration in the inlet sample was 6.278 ± 1.006 mg/L, whereas the phosphate concentrations at the CC and SC outlets were 5.071 ± 0.631 mg/L and 5.416 ± 1.038 mg/L, respectively. Phosphate is a soluble compound in water; thus, adsorption and chemical attachment on the media surface caused by different affinities are possible mechanisms in ISSF [[43], [44], [45], [46]].

**Fig. 2:**
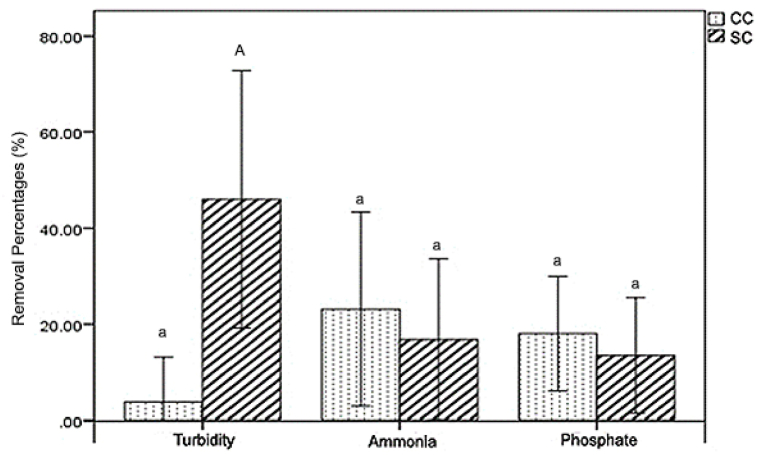
Effect of filter media on turbidity, ammonia, and phosphate removal percentage. Different letters (a-A) above the graphs indicate significant differences between the CC and SC mediums for the same parameter.

According to the findings presented in Fig. 2, the turbidity removal percentage achieved using CC media was determined to be 3.79 ± 9.35 %, whereas the turbidity removal percentage obtained using SC media was found to be 45.99 ± 26.84 %. Previous research also reported that SSF was superior in terms of turbidity removal, reaching up to more than 90 % in acclimatized and steady-state conditions [47,48]. Good removal of turbidity in SSF is facilitated by tight pores formed by biofilm-grown medium, which retain suspended particles [47,48]. The CC medium had a percentage of ammonia removal of 23.12 ± 20.2 %, whereas the SC media exhibited a percentage of ammonia removal of 16.77 ± 16.8 %. The CC media exhibited a phosphate removal rate of 18.03 ± 11.96 %, whereas the SC media had a phosphate removal rate of 13.48 ± 12 %. Low removal of turbidity in CC might be caused by the bigger gaps between media particles (discussed in Section 3.3), creating a bigger pore and causing particles that cause turbidity to pass on [49]. In the SC reactor, sand filled the gaps between the blood clam shell grains, causing tighter gaps between media particles, which then performed better at retaining turbidity particles [48]. Higher removal of ammonia and phosphate were subjected to the adsorption process caused by the clam shell medium. In CC, the portion of clam shell is higher than in SC. Blood clam shell is reported to have a positive surface charge [50], which is subjected to perform adsorption onto the media surface and pores via spontaneous adsorption mechanism [51] and calcium phosphate formation [52] (discussed further in Section 3.3). High phosphate removal was also reported by Xiong et al. [45] using clam shells, facilitated by the clam shells’ pores and different affinities. Calcination of clam shells elevated the phosphate removal by providing bigger pores for phosphate particles to attach and higher different affinities. The effects of medium type on the removal percentage of turbidity, ammonia, and phosphate are illustrated in Fig. 2.

### 3.2 The influences of acclimatization period on pH, and turbidity, ammonia, and phosphate removals

**Fig. 3:**
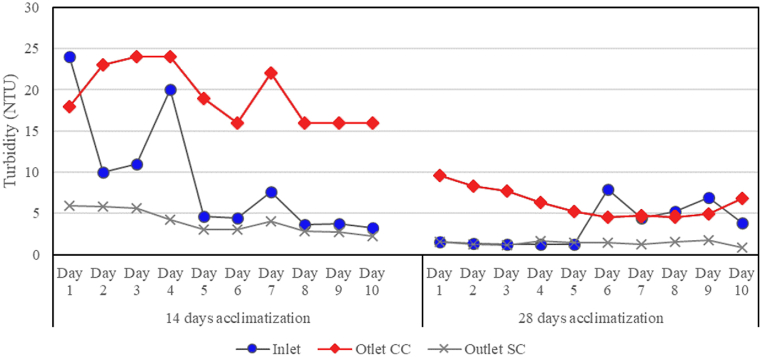
Effect of acclimatization period to turbidity readings.

Turbidity significantly decreased after 28 days of acclimatization. After a period of 14 days of acclimatization, it was observed that the turbidity value of the CC media outlet was greater compared to the inlet. This variation suggests that the system had not yet reached a condition of equilibrium, as evidenced by the discharge of the CC medium in the effluent. Overall, the turbidity value of CC medium showed a higher value than the inlet for both systems, but the value decreased on day 6 on the system with 28 days of acclimatization. The SC system showed a more stable value always below the inlet (Fig. 3). Future research should accommodate the analysis of the steady state of the ISSF before performing further parameter analysis [53]. In addition to the analysis of steady state, it is also suggested to analyze the breakthrough period of the medium (in which 100 % clogging of the medium occurs) to observe the required backwash time for the ISSF [21,54].

**Fig. 4:**
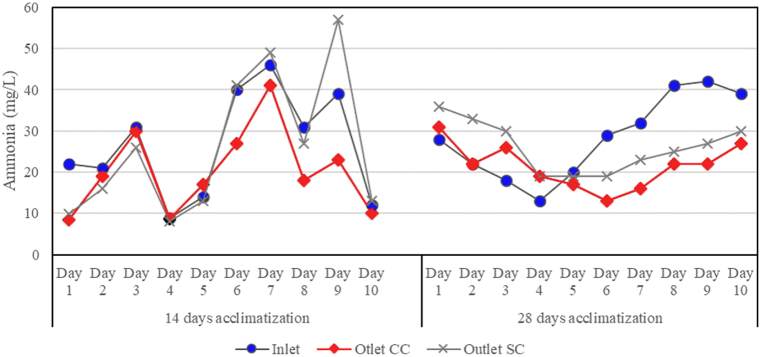
Effect of acclimatization period to ammonia readings.

**Fig. 5:**
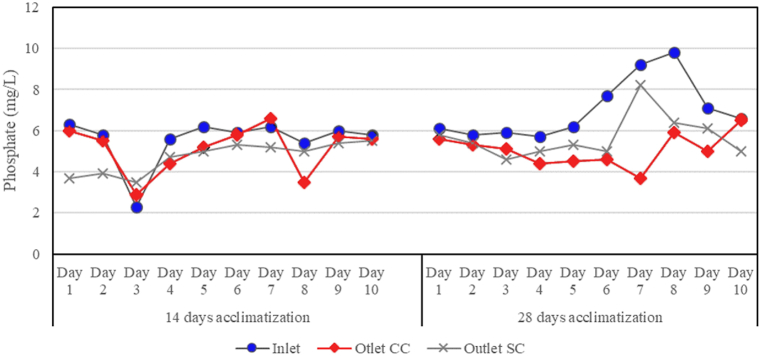
Effect of acclimatization period to phosphate readings.

Fig. 4 shows that the concentration of ammonia fluctuated in both systems. The 14-day acclimatization system showed a slight decrease in ammonia in both mediums, while an increase in concentration compared to the inlet occurred on the SC reactor on days 7 and 9 of running. During the run, there was an increase in pH from 7 to 8.55 in the SC reactor, and this might cause the NH_3_ and NH_4_^+^ speciation destabilization inside the system, causing the ammonia reading to be higher [55]. A similar result was obtained for phosphate concentrations (Fig. 5), in which the concentration fluctuated. Overall readings of phosphate in both acclimatization periods showed values lower than inlet, and 28 days of acclimatization showed a higher value of reduction based on the daily readings. A small portion of phosphate removal can also be subjected to the uptake by microorganisms to produce new cells and adsorption onto the medium surface [[56], [57], [58]].

**Fig. 6:**
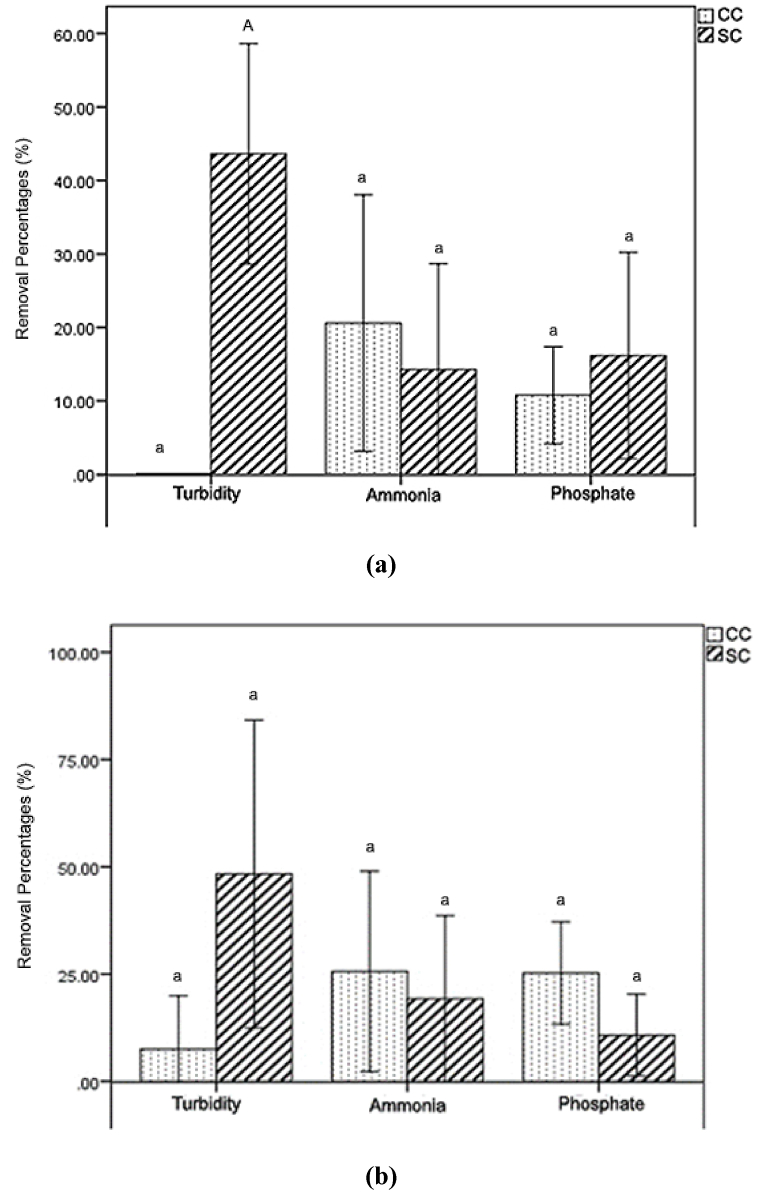
Removal of parameters on (a) 14 days and (b) 28 days of the acclimatization period. Different letters (a-A) above the graphs indicate significant differences between the CC and SC mediums for the same parameter.

Referring to the tabulation of parameter removal (Fig. 6), SC significantly outperformed CC in terms of turbidity removal. This result was obtained due to the tighter gaps between medium particles, which may result in better retention of turbidity particles in the filter. For SC, the 14 d acclimatization period showed a higher removal of turbidity (Fig. 6a). Ammonia removal was slightly higher in the CC reactor, which was due to the adsorption mechanism caused by the surface affinity of the CC medium [59]. In both medium types, the 28 d of acclimatization period gave a slightly higher value for ammonia removal (Fig. 6b). Phosphate removal was slightly higher for 28 days of acclimatization in CC medium, while significantly lower in SC medium as compared to the 14 days of acclimatization. This result might be obtained due to the accumulation of biofilms in SC pores, causing a reduction of oxygen transfer inside the system and creating slightly anoxic conditions that cause the phosphate-accumulating bacteria to release orthophosphate rather than storing it as poly-P [[60], [61], [62]]. Observations also showed that the schmutzdecke layer was formed after 14 days of acclimatization.

Effluent comparisons with the Indonesian national standard for domestic wastewater discharge (Peraturan Menteri Lingkungan Hidup Dan Kehutanan Republik Indonesia No. 68 Tahun 2016) are tabulated in Table 1. Referring to Table 1, the effluent of all system pHs was in the range of the standard. However, the CC a 14 d acclimatization period showed an effluent turbidity value higher than 9 NTU. In all systems, the effluent ammonia and phosphate values were exceeding the limit. Slightly higher than the ammonia standard was obtained by CC 14 d acclimatization systems, while slightly higher than the phosphate standard was obtained by SC with both acclimatization periods. Observing these results, optimization of the media composition, especially for SC, can be conducted to obtain a higher performance of ISSF to produce effluent below the standard value, especially for ammonia and phosphate parameters.

**Table 1:**
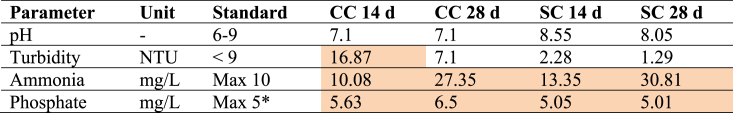
Effluent comparisons with national standard *Based on selective Class IV effluent standard; the highlighted value exceeds the national standard.

Compared with other research (Table 2), the currently presented result is considered average. Mažeikienė [63] reported the performance of a small-scale filtration unit treating domestic wastewater with 99.4 % suspended solids, 99.6 % ammonia, and 91.8 % total phosphorus removals. Kaetzl et al. [27] utilized biochar as an alternative filter media, which resulted in 31.1 % removal of turbidity, 7.1 % of ammonia, and 33.8 % of total phosphorus from sewage. Other than domestic wastewater, SSF can also be used to treat lake and rainwater, showing the removal of 89 % ammonia and up to 99 % turbidity, respectively [64,65]. Further optimization of this research can be conducted using models [64,66] focusing on some influential factors such as operating pH, hydraulic retention time, filter depth, and grain size [67] to obtain higher removal performances. On the other hand, additional parameters focusing on E. coli and total coliform removals by SSF might be interesting to add since SSF was reported to be efficient in removing coliforms [68].

**Table 2:** Comparison with previous studies.

Type of filter	Media	Treated wastewater	Removal	Reference
Intermittent slow sand filter (SC with 14 days acclimatization)	Sand and clam shells	Domestic	Turbidity 45.9 % Ammonia 16.7 % Phosphate 19.4 %	This research
Intermittent slow sand filter	Sand	Domestic	Total coliform 99 %	[68]
Adsorption filter	Filtralite	Domestic	Suspended solid 99.4 % Ammonia 99.6 % Total phosphorus 91.8 %	[63]
Slow sand filter	Biochar	Sewage	Turbidity 31.1 % Ammonia 7.1 % Total phosphorus 33.8 %	[27]
Slow sand filter	Sand	Contaminated lake	Ammonia 89 %	[64]
Slow sand filter	Sand	Rainwater	Turbidity 99 % Ammonia 95 %	[65]

### 3.3 SEM-EDX analysis

**Fig. 7:**
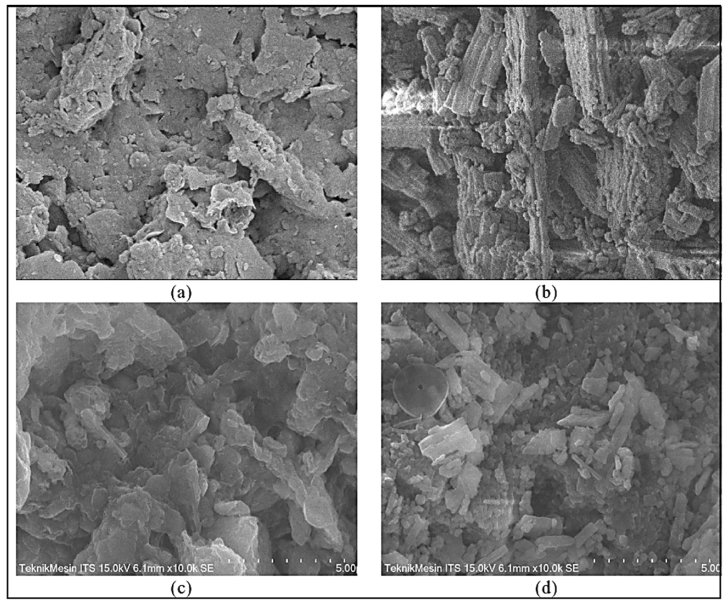
SEM images of (a) Sand media [SC], (b) Blood clam shell media [CC], (c) SC after 28 days, and (d) CC after 28 days of acclimatization.

Based on the SEM analysis, it can be concluded that the surface morphology of sand media (SC) before acclimatization at a 10,000 × magnification was wavy and that its edges were irregular, with pore sizes varying between 0.1 μm and 10 μm (Fig. 7a). The crushed blood clam shells (CC) exhibited grains with distinct sharp and sturdy edges, accompanied by many small cavities and larger pore gaps (Fig. 7b). After 28 days of acclimatization, SEM images show a change in the morphology of the sand and blood clamshell media. Both mediums showed duller surfaces, almost silk-like features, with decreasing size of gap pores. Although the surface of clam shell medium still looks coarser (Fig. 7d) as compared to the surface of sand medium (Fig. 7c), each appearance was quite similar. The silk-like features were an indication of biofilm forming on the medium surface [69], which can be associated with mucosa [70], which grew during the acclimatization and filled the gaps in medium pores and surface cavities. In this research, only biofilm analysis was conducted, while further study should also accommodate the analysis of schmutzdecke formation, focusing on its thickness over time and the changing microbial communities inside it [65,66]. Schmutzdecke formed as the result of microbial development after attachment to the very top of filter medium. Schmutzdecke acts as another layer of filter while also performing some biochemical processes while the pollutant passes through. Schmutzdecke is actually biofilm; however, it can have a thickness of a few millimeters up to 2 cm due to the accumulation and deposition on the top of the filter medium.

Table 3 shows the two different elemental compositions between sand and blood clam shell media. The sand media has a higher percentage of O, Na, Mg, Al, Si, K, Fe, and Ti, while the blood clam shell media has a higher percentage of C and Ca. The presence of Mg, Al, and Fe on the sand media surface may facilitate the adsorption of ammonia and phosphate onto the surface medium due to the formation of aluminium oxide [71] or magnesium iron hydroxycarbonate [72]. On the other hand, the presence of Ca also performed well in the removal of ammonia and phosphate via a spontaneous adsorption mechanism caused by surface affinity [51]. Adsorption of ammonia in the blood clam shell surface media also occurred due to the combination of protonation in water associated with Ca ions [73], while adsorption of phosphate was facilitated by the formation of calcium phosphate induced by different ionic strengths [52].

**Table 3:** Elemental composition of media based on EDX analysis.

Element	Sand (%)	Blood clam shell (%)
C	8.66	16.21
O	47.76	50.91
Na	1.11	0.38
Mg	0.66	0.0
Al	6.89	0.16
Si	23.63	0.03
K	1.62	0.03
Ca	2.00	31.83
Fe	6.94	0.47
Ti	0.72	0.0

## 4 Conclusion

The use of an intermittent slow sand filter (ISSF) with a combination of sand and blood clam shell media (SC) showed significantly higher removal of turbidity (3.79 % vs. 45.99 %), while only clam shell media (CC) showed better removal of ammonia (23.12 % vs. 16.77 %) and phosphate (18.03 % vs. 13.48 %). The use of two different acclimatization periods affects the removal of parameter regimes in ISSF. With 14 d of acclimatization, SC outperformed CC in terms of turbidity and phosphate removals, while in the 28 d of acclimatization, CC outperformed CC in terms of ammonia and phosphate removals. The differences in system regimes were due to the steady-state condition and stability of the biofilms inside the ISSF systems, which were exhibited by the scanning electron microscopy after 28 d of acclimatization.

## Data availability statement

Data will be made available upon request.

## CRediT authorship contribution statement

**Nurina Fitriani:** Supervision, Resources, Methodology, Funding acquisition, Conceptualization. **Ledy Theresia:** Investigation, Formal analysis, Data curation. **Timothy Tjahja Nugraha O'Marga:** Investigation, Formal analysis, Data curation. **Setyo Budi Kurniawan:** Writing – review & editing, Writing – original draft, Visualization, Validation, Data curation. **Agus Supriyanto:** Supervision, Resources, Funding acquisition. **Siti Rozaimah Sheikh Abdullah:** Writing – original draft, Validation. **Luuk C. Rietveld:** Writing – original draft, Supervision, Data curation.

## Declaration of competing interest

The authors declare that they have no known competing financial interests or personal relationships that could have appeared to influence the work reported in this paper.
